# Rapid Monitoring of the Stress Responses and Toxicity in Green Microalgae Cultures Using Pulse-Amplitude Modulated (PAM) Fluorometry

**DOI:** 10.3390/microorganisms13122712

**Published:** 2025-11-28

**Authors:** Vuk Radojicic, Aleksandra Skrobonja, Zivan Gojkovic

**Affiliations:** Vinča Institute of Nuclear Sciences, University of Belgrade, 11351 Belgrade, Serbia; vuk.radojicic@vin.bg.ac.rs (V.R.); aleksandra.skrobonja@vin.bg.ac.rs (A.S.)

**Keywords:** pulse-amplitude modulated fluorimetry, microalgae, photosynthetic efficiency, culture health, pollutants toxicity

## Abstract

Green microalgae are widely used as model organisms in ecotoxicology due to their sensitivity to environmental stressors and their critical role in aquatic ecosystems as primary producers at the bottom of the food web. Pulse-Amplitude Modulated (PAM) chlorophyll fluorometry is a non-destructive, rapid and informative method for assessing photosynthetic efficiency and culture health, particularly through parameters such as the maximum photochemical activity of PSII (F_v_/F_m_) and effective PSII activity (Φ_PSII_). Despite the growing number of studies utilizing PAM as an indicator rather than as a direct tool to evaluate microalgal stress responses, there remains a lack of standardized, accessible data for these key photosynthetic indicators. In this review, we analyze 38 sources, including 35 original research articles and 3 foundational references, to compile and compare reported values of F_v_/F_m_ and Φ_PSII_ across various green microalgae species exposed to a wide range of chemical and environmental stressors. We highlight species-specific differences in sensitivity, identify underrepresented contaminants such as ionic liquids and artificial sweeteners, and emphasize the need for systematic numerical reporting in future research. PAM is an excellent and reliable technique for rapidly assessing culture health of green microalgae and their photosynthetic performance in various culture conditions and the vast array of chemical and physical stressors.

## 1. Introduction

In recent decades, green microalgae have emerged as indispensable model organisms for environmental monitoring and biotechnology, due to their rapid growth, ecological adaptability, and sensitivity to chemical and physical stressors. They are among the most widely used photosynthetic microorganisms in ecology and blue biotechnology as they combine fast growth and high stress tolerance, and can generate valuable biomass and metabolites when cultivated in simple mineral media [1,2]. Green microalgae are predominantly used in industrial biotechnology, especially in agriculture, environmental protection, biofuels, biofertilizers, bioremediation, waste management and geomicrobiology [1,3]. Their unique photosynthetic machinery, centered on chlorophyll-containing photosystems located in thylakoids of the chloroplast, makes them highly responsive to environmental changes, and the responses can be quantitatively assessed using chlorophyll fluorescence techniques. Among these, Pulse Amplitude Modulated (PAM) fluorometry has become a standard non-invasive method for rapidly evaluating photosynthetic performance, particularly through indicators such as maximum photochemical PSII activity (hereafter referred to as maximum PSII activity; F_v_/F_m_) and effective PSII activity (Φ_PSII_) [4]. These parameters serve as reliable proxies for culture health, indicating potential cellular stress, photoinhibition, and stress recovery potential. Even though effective and maximum PSII activities are not fixed numerical values and are often used as approximate indicators of PSII performance, changes in these parameters provide valuable information for monitoring microalgal culture health.

Oxygenic photosynthesis is the primary biological source of atmospheric oxygen, driven by terrestrial vegetation and aquatic phototrophs such as phytoplankton, including microalgae and cyanobacteria [5]. Phytoplankton alone contributes an estimated 50–80% of global oxygen production, underscoring its central role in the Earth’s oxygen cycle [6,7]. Because of this importance, monitoring photosynthetic efficiency and stress responses in microalgal communities is essential for evaluating their resilience to environmental stressors such as climate change and pollution as well as overall aquatic ecosystem health.

With the increasing presence of pharmaceuticals, pesticides, heavy metals, nanoparticles and emerging contaminants such as ionic liquids in aquatic environments, the need for robust and sensitive bioindicators is growing. Green microalgae, especially genera such as *Chlorella*, *Scenedesmus* and *Chlamydomonas*, are widely used to assess the toxicity of diverse environmental stressors. However, despite the expanding ecotoxicological literature, a major gap remains in the availability and organization of quantitative fluorescence data. Many studies do not report numerical values for key photosynthetic indicators or provide them only in graphical form, which limits comparability and reduces reproducibility. To address this issue, the present review compiles data from 35 original research articles and 3 foundational theoretical references to provide a comparative overview of reported PSII-related parameters in green microalgae under chemical and environmental stress. Only studies that included exact numerical values, percentage changes or extractable figure-based data were considered. Our analysis focused on two commonly reported PAM fluorometry parameters (F_v_/F_m_ and Φ_PSII_), used to monitor stress responses across a range of microalgal species. The compiled data reveal patterns of species-specific sensitivity and highlight the need for more systematic reporting of core physiological metrics. Additionally, establishing a standardized reference dataset for F_v_/F_m_ and Φ_PSII_ under defined stress conditions would improve data interpretation and support species selection for biotechnological and environmental applications.

## 2. Basic Principles of the Pulse Amplitude Modulated (PAM) Fluorescence of Chlorophyll Measurements in Photosynthetic Microorganisms

Green microalgae contain the photosynthetic pigments chlorophyll *a* and *b*, which enable the capture of light energy and its conversion into chemical energy, available for cellular processes [8]. This light-driven conversion occurs in the thylakoid membranes, primarily through two major protein-pigment complexes: Photosystem I (PSI) and Photosystem II (PSII). These complexes are linked in a series of oxidative-reduction reactions that transfer electrons uphill from water to the final acceptor, NADP^+^ [9]. Together, PSI and PSII drive the light-dependent reactions of photosynthesis, supporting cellular energy metabolism and growth.

There are three main de-excitation pathways for photons absorbed by PSII: photochemistry (photochemical quenching), fluorescence, and heat dissipation (non-photochemical quenching (NPQ)) [8,10,11]. Chlorophyll fluorescence reflects the balance between photochemical and non-photochemical energy dissipation, and although it accounts only for 0.5–10% of total absorbed light energy, it is a sensitive and informative indicator of photosynthetic activity and stress responses [10,11]. Both PSI and PSII emit fluorescence, but the detectable signal originates predominantly from PSII. The contribution of PSI is typically disregarded in standard fluorescence measurements, although it can become significant under specific conditions, in which case it cannot be neglected [4,12]. The principles of PAM fluorometry and the saturation pulse method are described in detail in [11,12,13,14].

The PAM technique provides information on the distribution of absorbed light energy between photochemical and non-photochemical processes in PSII [15]. Photons absorbed by PSII associated chlorophylls can drive photochemistry through electron transfer from the reaction center P680, to the primary quinone acceptor of PSII, Q_A_. Alternatively, absorbed light energy may be dissipated as chlorophyll fluorescence or heat [16]. These three processes (photochemistry, chlorophyll fluorescence, and heat loss) compete for the same excitation energy [16]. During dark adaptation, PSII reaction centers progressively relax to an open, oxidized state. In this state, non-photochemical processes are minimized, Q_A_ is fully oxidized and no proton gradient is present across the thylakoid membrane [12]. The baseline fluorescence measured after prolonged dark adaptation (typically around 10 to 15 min) is defined as minimal fluorescence, F_0_. F_0_ is generally measured using low frequency, pulse-modulated measuring light (10–50 Hz; <1 µmol_photons_ m^−2^ s^−1^), ensuring that no reduction of PSII acceptors occurs [13]. Due to the very low light intensities used to measure F_0_ (0.05 µmol_photons_m^−2^s^−1^), it can be measured continuously without compromising the dark state [12]. A strong saturating pulse with photosynthetic photon flux density PPFD > 10 000 µmol_photons_m^−2^s^−1^, and a duration of 0.4 to 0.8 s is sufficient to fully reduce Q_A_ and close all reaction centers [4,17,18]. Microalgal exposure to such short, intense pulses saturates PSII reaction centers as excitation is rapidly funneled through the light-harvesting complexes to PSII and PSI.

A PSII reaction center is considered closed when Q_A_ is in the reduced state (Q_A_^−^), preventing further charge separation. Because the redox state of Q_A_ cannot be measured directly, Chl *a* fluorescence is used as an indirect proxy [11]. Closure of reaction centers reduces photochemical efficiency and enhances the proportion of energy lost through alternative relaxation pathways, including fluorescence. Open reaction centers act as fluorescence quenchers, and fluorescence levels increase proportionally with the degree of PSII reduction, saturation and closure [4], a process known as photochemical quenching. In contrast, non-photochemical quenching is not directly related to photochemistry and represents heat dissipation of absorbed energy. This mechanism protects PSII reaction centers from damage caused by high or fluctuating irradiance [4].

**Figure 1:**
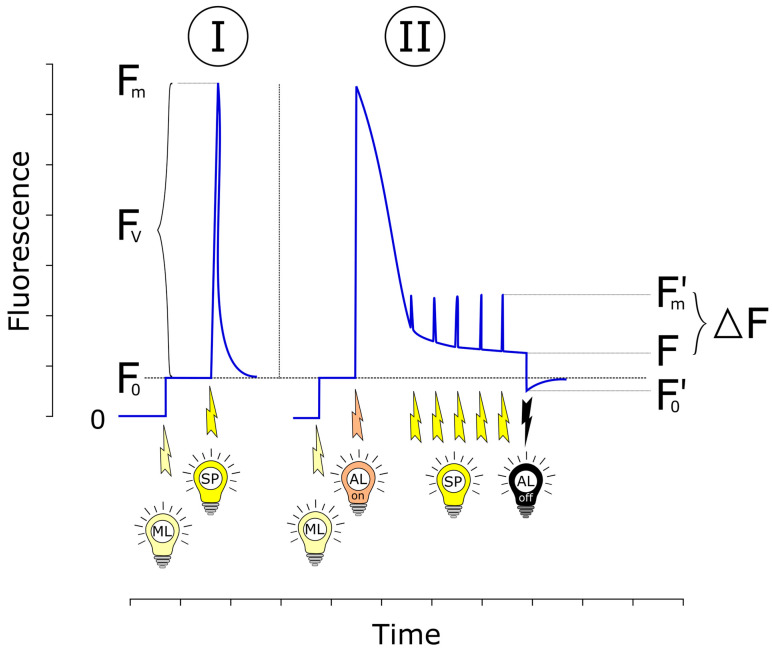
Schematic representation of the pulse amplitude modulation (PAM) method and the main fluorescence parameters (adapted from [8,10,12]). Maximum fluorescence level (F_m_) and zero fluorescence level (F_0_) are measured in the dark-adapted sample (with all reaction centers in the open state), using modulated measuring light (ML) followed by the saturating light pulse (Phase I). Then, the sample is illuminated with actinic light, which is able to drive photosynthesis and a series of saturating pulses to reach steady (light-adapted) state fluorescence (F′) and steady state maximum fluorescence F′_m_ level. Finally, the actinic light (AL) and saturating pulses (SP) are switched off to measure the F′_0_ level of the light-adapted sample (Phase II). To measure zero fluorescence in the light phase (F′_0_), the sample has to be darkened and Q_A_ has to be rapidly fully oxidized. To achieve that, some instruments apply far-red light (735 nm) to enhance the selective excitation of PSI and facilitate oxidation of the electron transport chain [12].

The maximum fluorescence measured when all PSII reaction centers are fully reduced and closed is designated as F_m_. Together with minimal fluorescence (F_0_), this value is used to calculate F_v_/F_m_, widely recognized as an indicator of PSII photochemical efficiency and overall culture health in green microalgae. Variable fluorescence (F_v_) is defined as the difference between F_m_ and F_0_ in dark-adapted samples (Figure 1). The F_v_/F_m_ ratio provides a reliable measure of the physiological status and maximum photochemical efficiency of PSII [8,10,17].

(1) $$F_{v}/F_{m}=(F_{m}-F_{0})/F_{m}$$

Furthermore, F_v_/F_m_ reflects the intrinsic or maximum capacity of the PSII system and allows non-invasive assessment of photosynthetic performance in microalgae. It is therefore widely used as an indicator of microalgal physiological status, with values typically ranging between 0.6 and 0.8 in healthy green microalgae cultures [4]. However, this optimal range can vary depending on light conditions, physiological state and exposure to chemical or environmental stressors [4,8].

Because of its sensitivity and ease of measurement, F_v_/F_m_ is a robust parameter for monitoring microalgal health and for evaluating the effects of physical and chemical stressors. When dark adaptation is not feasible or when maximum photochemical efficiency is not the primary parameter of interest, fluorescence can instead be measured under light-adapted conditions. In this approach, steady-state fluorescence (F′) is recorded immediately before a saturating light pulse, and maximum fluorescence in the light-adapted state (F′_m_) is measured immediately after the pulse. These relative values are used to calculate the Effective quantum efficiency of PSII (Φ_PSII_), expressed as ΔF/F′_m_, where ΔF is the difference between F′_m_ and F′. Φ_PSII_ represents the fraction of absorbed photons that drives electron transport in PSII and provides real-time information on PSII photochemical performance under ambient or experimental light conditions.

(2) $$\Phi_{PSII}=\frac{\Delta F}{{F^{\prime }}_{m}}=({F^{\prime }}_{m}-F^{\prime })/{F^{\prime }}_{m}$$

Fluorescence parameters of both dark-adapted and light-adapted states can be used to calculate further important quenching parameters: q_P_—coefficient of photochemical quenching; q_N_—coefficient of non-photochemical quenching; NPQ—non-photochemical quenching parameter.

All valuable photochemical parameters can be obtained using the simple and non-destructive PAM technique [5]. NPQ is an efficient process that protects PSII reaction centers from photoinhibition [19]. According to the current understanding, the dominant component of NPQ is energy-dependent quenching (qE), which arises from the combined effects of lumen acidification, xanthophyll cycle activity, and conformational changes in PSII antenna proteins (Lhcb and PsbS) [15,19,20]. NPQ is triggered by the proton gradient across the thylakoid membrane, either through direct protonation of antenna components or indirectly via the xanthophyll cycle [19]. The difference between F_m_ and F′_m_ can also be used to quantify NPQ [18].

Φ_PSII_ is a useful parameter for quantifying the proportion of absorbed light used in photochemistry under ambient conditions, but it is more sensitive to short-term environmental fluctuations than F_v_/F_m_. A representative example is the daily variation of Φ_PSII_ in outdoor cultivated green microalgae [4,8]. Values are typically higher in the early morning, when most PSII reaction centers are open, and decrease by midday due to partial reaction center closure caused by prolonged high light exposure [4,8]. Because of these characteristics, Φ_PSII_ generally exhibits lower absolute values than F_v_/F_m_ (Figure 1). PAM fluorometry also enables indirect estimation of the relative electron transport rate (rETR), which is derived directly from Φ_PSII_ [21]. rETR through PSII is calculated using the following equation [10,22,23]:

(3) $$rETR=\Phi_{PSII}\cdot PAR\cdot 0.5$$

where PAR is the actinic photosynthetically active radiation of the AL used.

The rETR parameter is less commonly used for comparing photosynthetic efficiencies among microalgae because it is strongly influenced by photobioreactor design and culturing conditions. Since rETR depends directly on light availability, values can differ substantially between indoor cultures with controlled illumination and outdoor systems exposed to variable light regimes [4,15]. For example, even in diluted outdoor open pond cultures with biomass concentrations near 0.6 g_DW_/L, light penetration is very limited and the effective photic layer is only 1 cm [15]. In such large, continuously mixed systems, NPQ relaxation may also be too slow to fully counteract the rapid transitions between light and dark zones experienced by individual cells [15]. Because of these constraints, the present review focuses primarily on the more consistently reported F_v_/F_m_ parameter when evaluating the effects of various stressors on microalgal cultures.

An alternative way to assess photosynthetic efficiency is through the parameter alpha (α), which is also proportional to the maximum PSII activity and defined as the initial slope of the photosynthesis rate vs. irradiance (P–I) curve in its exponential region [17]. The P–I curve is derived from measurements of oxygen evolution (or, in some cases, CO_2_ fixation) across a gradient of light intensities. Steady-state P–I curves typically consist of three zones: photolimitation zone, where photosynthetic activity increases linearly with irradiance; photosaturation zone, where oxygen production reaches a plateau; and photoinhibition zone, where excessive irradiance causes a decline in oxygen production (harmful zone) [17,18]. The slope of the curve in the photolimitation zone represents α and reflects the efficiency of light utilization under sub-saturating light conditions, providing an estimate of the maximum efficiency of light conversion into biomass [17].

Rapid light response curves (RLCs) are generated by measuring fluorescence responses under 8–12 different actinic lights (ALs) steps with progressively increasing PAR levels [24]. Although visually similar to the steady-state P-I curves, RLCs describe the relationship between relative electron transport rate (rETR) and irradiance [18]. They provide insight into electron transport saturation and the short-term photophysiological performance of microalgal cultures [18]. During RLC measurements, each AL incubation step typically lasts 10–30 s before a saturation pulse is applied to determine the rETR at that irradiance level [18]. The resulting curve plots rETR as a function of actinic irradiance. The RLC provides three important parameters: the initial slope α (equivalent to that of the P-I curve), ETR_max_ or the maximal electron transport rate in PSII at minimum saturating irradiance—Ik [24,25]. The Ik value is determined from the intersection of the α slope with the ETR_max_ level [18]. Ik can be used as the index of light adaptation of PSI or PSII and is expressed as [26]:

(4) IK=ETRmaxα

While α is a highly informative indicator of microalgal health and photosynthetic performance, its labor-intensive and time-consuming nature makes it less commonly reported than F_v_/F_m_ or Φ_PSII_. Nonetheless, its relevance for characterizing photosynthetic efficiency remains significant. Another useful fluorescence parameter that describes the overall photosynthetic capacity of the cell is the functional absorption cross section of PSII, (σPSII), derived from flash-induced fluorescence saturation curves generated using single-turnover flashes [13].

In addition to PAM fluorometry, rapid fluorescence induction or relaxation kinetics provide alternative approaches for assessing photosynthetic performance [18]. Whereas PAM focuses on the distribution of absorbed energy between photochemical and non-photochemical processes, rapid fluorescence induction traces the redox status of the PSII electron transport chain [15,18]. Some fluorometers are able to construct fast fluorescence rise kinetics curve (Kautsky test or OJIP curve), which contains detailed insights into the functional state of the photosynthetic apparatus [15,27]. The polyphasic OJIP curve is time-resolved and consists of four characteristic phases (O, J, I and P) each reflecting a distinct redox state of the PSII electron acceptors in time (ms) [27]. Dark-adapted samples are firstly exposed to weak ML and then to strong AL, when fluorescence rises rapidly from the origin (O) to a peak (P), via 2 inflections (J and I), in less than 1 s [18]. For example, point O corresponds to the minimal Chl *a* fluorescence yield with all PSII reaction centers open (dark-adapted state), points J and I reflect the light-driven reduction of primary quinone receptors Q_A_ and Q_B_, corresponding to the inverse of PSII turnover rate [13,27]. Finally, the peak of the fluorescence is recorded at P level, the maximal fluorescence yield (F_m_) is reached when the plastoquinone (PQ) pool becomes fully reduced [28]. OJIP parameters are often used to explain the energy fluxes through PSII, but the complexity of their interpretation limits their application in rapid toxicity screening of microalgal cultures [28]. Detailed interpretation and description of the OJIP curve can be found elsewhere [27].

Description of additional photosynthetic parameters, measurable with various fluorometers, is out of the scope of this review and is available elsewhere [11,12,13,14,29]. It is also crucial to emphasize that none of the fluorescence parameters obtained by PAM fluorometry are absolute in value. Even within a single culture, they may vary spatially and temporally. However, they offer critical insight into the photosynthetic apparatus and reflect both the intrinsic physiological status of the culture and its responses to environmental or experimental stimuli.

Measurement of the absolute ETR enables fluorescence efficiencies to be converted into quantitative rates of electron transport [30]. Unlike rETR values derived from chlorophyll fluorescence (Equation (3)), absolute electron transport rate (ETR) reflects the true rate of electron flow through the photosynthetic electron transport chain. Its determination requires knowledge of the already mentioned functional absorption cross-section of PSII (σPSII, also known as the sample absorptance), and the PSII to PSI absorptivity ratio, to convert fluorescence efficiencies into quantitative rates of electron transport [31,32,33]. Absolute ETR per open PSII reaction center is calculated as the product of the optical absorption cross section of PSII (σPSII^opt^), light intensity and the effective quantum efficiency of PSII (Φ_PSII_), where σPSII = σPSII^opt^ × Φ_PSII_. This value is then multiplied by the fraction of dynamically open reaction centers, expressed as the ratio of variable fluorescence under a given irradiance (ΔF′_v_) to the maximum variable fluorescence (F′_v_) at this irradiance [32].

(5) $$ETR=\sigma PSII\cdot E\cdot \frac{{∆F^{\prime }}_{V}}{{F^{\prime }}_{V}}$$

Absolute ETR quantifies the number of electrons transported per reaction center per unit time and provides a mechanistic link between photochemical efficiency and overall photosynthetic energy conversion [31,32]. A major limitation of this approach is that it requires prior determination of the concentration of functional PSII reaction centers, which has restricted the routine use of FRR fluorometers for estimating electron transport rates [18]. Technical details regarding instrument design, illumination types (SL, ML, AL), wavelength settings and operational modes can be found in the respective manufacturers’ documentation. Furthermore, while PAM fluorometry is a robust and widely used technique, its parameters should be interpreted carefully and, when possible, verified using complementary methods to avoid artifacts related to dark adaptation or culture-specific optical properties. Because fluorescence-based estimates of photosynthetic efficiency correlate to quantum yields of other photosynthetic processes, such as O_2_ evolution and CO_2_ fixation, PSII activity can be verified in the lab using, for example, Clark’s electrode and O_2_ evolution measurements [18].

## 3. Green Microalgae Sensitivity to Different Stressors Reflected in Their Photosynthetic Activity and Maximum PSII Activity

Green microalgae display extensive diversity in habitat, morphology and physiology, which leads to different responses to identical environmental stimuli. These varied responses can be either detrimental or beneficial, depending on the stressor and species. Such heterogeneity in contaminant sensitivity poses a challenge for research and risk assessment, as each species must be individually evaluated to accurately predict its behavior under specific conditions. At the same time, this diversity provides a broad reservoir of microalgal strains with different tolerances, ecological niches and potential biotechnological applications.

*Ankistrodesmus* sp. has been investigated for its potential in biotechnological applications involving selenium nanoparticles (SeNPs), due to their effects on biomass and lipid production, as well as their ability to increase tolerance to cadmium (Cd), a heavy metal known for both growth-stimulating and toxic effects in microalgal cultures grown in recycled media [34]. Cd is a toxic and non-essential heavy metal with the ability to impair algal growth and photosynthesis even at low concentrations [35,36,37]. Cd toxicity in green microalgae can manifest through direct inhibition of PSII, induction of oxidative stress, pigment degradation, chloroplast damage and disrupted respiration. The addition of 15 μM Cd and 2 mg/L of SeNPs to *Ankistrodesmus* sp. cultures resulted in a 63% increase in lipid accumulation, along with increase in CO_2_ fixation, biomass yield, photosynthetic pigment concentration and nutrient removal efficiency [34]. Notably, the highest CO_2_ fixation rate (0.98 g/L/day) was observed in cultures treated with SeNPs alone. SeNPs also improved photosynthetic performance, increasing F_v_/F_m_ by approximately 8%. Moreover, when applied together with Cd, SeNPs mitigated metal toxicity, limiting the decline in F_v_/F_m_ to about 3% [34]. These findings indicate a promising synergistic role of SeNPs in enhancing productivity and stress resilience in *Ankistrodesmus* sp. However, the effects of nanoparticles vary considerably across types. Unlike SeNPs, nanoparticles such as Ag, TiO_2_ and ZnO have been reported to cause inhibitory or toxic effects in green microalgae, demonstrating that nanoparticle impact strongly depends on their composition, dose, and exposure time [38,39,40].

Green microalgae of the genus *Chlamydomonas*, particularly *Chlamydomonas reinhardtii*, are among the most extensively studied model organisms in genetics, proteomics and physiological studies in bioscience [41,42,43,44,45,46,47]. However, despite their value in laboratory studies, their application in pilot- and large-scale cultivation remains limited, although there are some improvements in this field [48,49,50]. This limitation comes primarily from their sensitivity to shear stress, mechanical pumping and other physiological constraints, typical of industrial-scale cultivation systems. Although efforts have been made to develop strains with improved resilience for large-scale application, these are still in early stages of development [15,51]. Among the reviewed studies, *C. reinhardtii* was the most frequently used species, especially in short-term (15 min to 24 h) toxicity assessments involving various stressors [28,41,52,53,54]. The impact of three endocrine disrupting compounds (4-octylphenol, 4-nonylphenol and b-estradiol) was studied in four microalgal model organisms: two green microalgae (*C. reinhardtii* and *Pseudokirchneriella subcapitata*), and two strains of the cyanobacterium *Microcystis aeruginosa* [28]. At ppm-level concentrations, *C. reinhardtii* exhibited a reduction in F_v_/F_m_ of 33%, 73% and 6% for 4-nonylphenol, 4-octylphenol and b-estradiol, respectively, making it the second most sensitive organism in the study, following one *Microcystis* strain [28]. In a separate study, *C. reinhardtii* showed a 25% decline in F_v_/F_m_ upon exposure to concentrations of aclonifen exceeding 0.1 mM, indicating its vulnerability to certain herbicides [41]. Another investigation compared the effects of cerium-oxide nanoparticles, both dispersed and agglomerated, with cerium nitrate (Ce(NO_3_)_3_) on C. *reinhardtii* [54]. Dispersed nanoparticles had no visible effect on the F_v_/F_m_, likely due to the precipitation of dissolved Ce^3+^ ions with phosphates, which limited their bioavailability. At similar concentrations, agglomerated nanoparticles showed a minor decrease in photosynthetic efficiency, while Ce(NO_3_)_3_ caused a substantial reduction, attributed to its higher solubility and bioavailability [54]. Selenate exposure further demonstrated sensitivity, with inhibition detected at concentrations as low as 2.4 µM, and a 66% reduction in F_v_/F_m_ at 9.3 µM [44]. *C. reinhardtii* has also been used in studies involving ionizing radiation and radionuclides. For instance, exposure to 1 mg/L of uranyl nitrate (UO_2_(NO_3_)_2_) for one hour resulted in a 25% decrease in F_v_/F_m_ [53]. Interestingly, both F_0_ and F_m_ were reduced by the exposure, leading to a less pronounced decrease in F_v_/F_m_ than expected [53]. In another study investigating the effects of gamma radiation, *C. reinhardtii* displayed relatively stable F_v_/F_m_ values, while Φ_PSII_ had a noticeable decline, indicating that PSII reaction centers were not damaged by the radiation [52], but electron transport downstream was impaired, consistent with the dependence of rETR through PSII on Φ_PSII_ and PPFD [4].

Among the studies reviewed here, the genus *Chlorella* was the most frequently investigated, reflecting its importance in microalgal biotechnology (see Table 1). Research encompassed a wide range of stressors, including pesticides, inorganic metals, ionic fluids, and nutrient deficiencies. The most commonly studied species were *C. vulgaris* and *Auxenochlorella pyrenoidosa* (older name *C. pyrenoidosa*), with fewer reports on *C. sorokiniana* and *Chlorella* sp. strains. Organic pollutants, particularly pesticides, were the most extensively examined group of stressors for the *Chlorella* genus. In *C. vulgaris*, exposure to 20 μg/L of carbofuran, diuron and methyl viologen resulted in F_v_/F_m_ decreases of 0.65%, 13.05% and 27.48%, respectively [27]. On the other hand, 20 μg/L of malathion had no visible effect on F_v_/F_m_. These declines in F_v_/F_m_ indicate impaired PSII efficiency, likely due to disruptions at the level of electron transport or reaction center structure. In *A. pyrenoidosa*, atrazine (10 μg/L) [30], azoxystrobin (2.5 mg/L) [55], diuron (10 μg/L) [30] and fuberidazole (0.2 mg/L) [30] reduced F_v_/F_m_ for 10%, 5.9%, 37.5% and 9.7% respectively. It was suggested that the duration of exposure to triazophos in *A. pyrenoidosa* cultures is time-dependent [56]. The cultures were exposed to varying concentrations of triazophos and monitored for longer periods, with a significant F_v_/F_m_ decrease after 48 h, but with the total recovery in the next 24 h, thus minimizing the impact of triazophos on microalgal photosynthetic activity [56]. A similar trend was reported in acidophile green microalga *Coccomyxa onubensis* exposed to 15 nM of methylmercury (MeHg), where initial F_v_/F_m_ values decreased from 0.71 to 0.53 during the first 24 h, and then gradually increased to 0.60 after 72 h of exposure, suggesting gradual culture adaptation to MeHg exposure [57].

In addition to commonly studied stressors, several other organic compounds have shown significant effects on photosynthetic efficiency in *Chlorella* species. For instance, benzoquinone-dibromothymoquinone, tested on *C. vulgaris*, exhibited a rapid inhibitory effect on PSII activity, although this effect dissipated quickly [27]. Similarly, exposure of *A. pyrenoidosa* to p-benzoquinone (5 mg/L), phenanthrene (10 μg/L), phenol (0.6 g/L), trichloroacetonitrile (2 mg/L), and trichloroacetonitrile uric acid (0.5 mg/L) led to reductions in the maximum PSII activity (F_v_/F_m_) of 31.2%, 15.1%, 18.3%, 14.5%, and 32.28%, respectively, within just 1 h of exposure [30].

A relatively novel and understudied class of emerging contaminants is ionic liquids, salts that are liquid at or near room temperature, often composed of organic cations (e.g., imidazolium derivatives) and various anions [58,59]. Due to their tunable physicochemical properties and growing industrial applications, some ionic liquids are now commercially available. However, their potential environmental toxicity remains insufficiently explored. For example, [C_12_mim]Cl significantly reduced the maximum activity of PSII in *Chlorella* sp. at concentrations as low as 12 μg/L, while the shorter-chain analogue [C_4_mim]Cl required considerably higher concentrations to produce a similar effect [60].

**Table 1:** Literature data of the PSII activity measurements and changes with different stressors in the cultures of green microalgae, in alphabetical order by the species name.

Algae	Stressor	Stress Conditions	F_v_/F_m_	F_v_/F_m_ Change	Duration of Exposure	Ref.
*Ankistrodesmus* sp.	Se NP	2 mg/L	0.79	7.94%	3 days	[34]
	Se NP + Cd	2 mg/L + 15 μM	0.73	0.0%	3 days	[34]
	Se NP	2 mg/L	0.623	−3.26%	7 days	[34]
	Se NP + Cd	2 mg/L + 15 μM	0.594	−7.76%	7 days	[34]
*Chlamydomonas reinhardtii*	SeO_4_^2−^	9.3 µM	0.58	−22%	24 h	[44]
	Aclonifen	0.1 mM	0.5	−25.0%		[41]
*Chlorella pyrenoidosa (Auxenochlorella pyrenoidosa)*	Ariazophos	1 mg/L	0.69	0.41%	96 h	[56]
	[C4mim]Cl	10 mg/L	0.518	−23.82	96 h	[60]
	[C12mim]Cl	0.012 mg/L	0.43	−37.68%	24 h	[60]
	Azoxystrobin	2.5 mg/L	0.6	−5.90%	4 days	[55]
	Diuron	10 μg/L	0.373	−37.52%	60 min	[30]
	Atrazine	10 μg/L	0.544	−10.08%	60 min	[30]
	Fuberidazole	0.2 mg/L	0.528	−9.74	60 min	[30]
	Phenanthrene	10 μg/L	0.511	−15.12%	60 min	[30]
	Phenol	0.6 g/L	0.485	−18.35%	60 min	[30]
	p-benzoquinone	5 mg/L	0.425	−31.23%	60 min	[30]
	Trichloroacetonitrile uric acid	0.5 mg/L	0.407	−32.28%	60 min	[30]
	Trichloroacetonitrile	2 mg/L	0.495	−14.51%	60 min	[30]
*Chlorella sorokiniana*	SeO_4_^2−^	212 μM	0.565	−20%	120 h	[61]
*Chlorella* sp.	N starvation		0.54	−37.93%	28 days	[24]
	P starvation		0.6	−28.57%	28 days	[24]
	Fe starvation		0.65	−22.62%	28 days	[24]
	Sudden transfer to demi water		0.67	−20.24%	28 days	[24]
*Chlorella vulgaris*	Diuron	20 μg/L	0.653	−13.05%	80 min	[27]
	Dibromothymoquinone	20 μg/L	0.616	−16.76%	60 min	[27]
	Methyl viologen	20 μg/L	0.549	−27.48%	150 min	[27]
	Malathion	20 μg/L	0.758	0.0%	30 min	[27]
	Carbofuran	20 μg/L	0.759	−0.65%	30 min	[27]
	Cu	1 μM	0.642	7.90%	72 h	[62]
	Cu	4 μM	0.493	−17.14%	72 h	[62]
	Light intensity	300 µmolm^−2^ s^−1^	<0.4	−60%	15 days	[63]
*Dunaliella salina*	Temperature	40 °C	0.37	−43.94%	4 h	[23]
	MeHg	15 nM	0.28	−58%	72 h	[57]
*Graesiella emersonii*	Chlorpyrifos	27.21 mg/L		−10.61%		[25]
	α-cypermethrin	14.7 mg/L		−24.30%		[25]
*Monoraphidium braunii*	HS1500	DOC 4.17 mM	0.759	4.11%		[64]
	HuminFeed	DOC 4.17 mM	0.757	11.65%		[64]
*Pseudokirchneriella subcapitata*	Metribuzin	40 nM	0.65	−8.31%	80 min	[65]
	Atrazine	460 nM	0.6	−13.86%	80 min	[65]
	Glyphosate	440 μm	0.634	−9%	80 min	[65]
	KCN	1 mM	0.64	0%	80 min	[65]
	Cr (VI)	41 µM	0.16	−84%	72 h	[66]
	Cu	1.3 µM	0.68	−32%	72 h	[66]
	Zn	2.5 µM	0.72	−28%	72 h	[66]
	Cd	1.9 µM	0.85	−15%	72 h	[66]
*Scenedesmus obliquus (Tetradesmus obliquus)*	HS1500	DOC 4.17 mM	0.714	1.71%		[64]
	HuminFeed	DOC 4.17 mM	0.757	2.02%		[64]
	Cd	3 mg/L	0.6	−13.52%	2 days	[67]
	Cd + acesulfame	3 mg/L + 1 mg/L	0.61	−13.51%	2 days	[67]
	Cd + sucralose	3 mg/L + 1 mg/L	0.69	0.72%	2 days	[67]
	S-(+)-IBU	10 mg/L	0.573	−11.57%	96 h	[68]
	rac-IBU	10 mg/L	0.577	−10.96%	96 h	[68]
	Aspirin	50 mg/L	0.138	−78.70%	96 h	[68]
	Ketoprofen	0.1 mg/L	0.596	−8.02%	96 h	[68]

Among inorganic stressors, metal ions and salts are common aquatic contaminants, often originating from natural geological sources and anthropogenic activities such as mining, agriculture and industrial discharge [69].

The growth-promoting role of the essential micronutrient Cu(II) in *C. vulgaris* is reversed as the concentration in the growth medium reaches EC_50_ values (effective concentration of the toxicant which induces 50% growth rate reduction) of 3.16 μM after 48 h exposure to Cu(II) [62]. F_v_/F_m_ was maximal at 1 μM Cu(II) but decreased to almost zero at 4 and 5 μM Cu(II) [62]. The authors suggested that the negative effect of high-dose Cu(II) on the F_v_/F_m_ may be due to Cu(II) harmful effects on the electron transport chain in PSII, indicating that the photo-damage of PSII may be accompanied by an increase in energy dissipation as heat and an increase in photo-damage to the photosynthetic apparatus [62]. A study on *C. sorokiniana* revealed that exposure to 238.2 μM of selenate (SeO_4_^2−^) reduced the rate of oxygen evolution by 50%, despite no significant changes in chlorophyll or carotenoid content [61]. The same study reported a 25% decrease in Φ_PSII_ for Se-exposed cultures compared to control [61]. Interestingly, both selenate and selenite added in sublethal doses induced partial disruption of the chloroplast ultrastructure, characterized by “fingerprint-like” rearrangement of thylakoid membranes and a reduction of normal grana stacking, resulting in a granular and less dense stroma [44,61,70].

With the growing importance of large-scale algal biomass production, monitoring physiological responses of microalgae to nutrient imbalances, deficiencies and physiological stress in outdoor cultivation systems has become a valuable approach for assessing culture health and optimizing productivity [71,72,73]. Several studies have examined the effects of selective nutrient deprivation on photosynthetic performance [24,74]. Progressive removal of individual macronutrients (iron, nitrate and phosphate) from the growth medium in *Chlorella* sp. cultures over a 28-day period resulted in reductions of 22.62%, 37.93%, and 28.57% in the maximum PSII activity, respectively [24]. Interestingly, the complete absence of all nutrients (i.e., sudden culture resuspension in distilled water) had a comparatively smaller effect on both F_v_/F_m_ and the initial slope of the P-I curve (α) than the selective removal of nitrate or phosphate. This unexpected result suggests that microalgae may activate distinct adaptive mechanisms under total nutrient starvation compared to selective macronutrient stress [24]. Although nutrient stress generally leads to a decrease in the F_v_/F_m_, the relationship between F_v_/F_m_ and growth rates is highly nonlinear. As a result, the authors concluded that it is impossible to quantify the reduction in phytoplankton growth rates using only F_v_/F_m_ [33]. Light intensity has also been examined as a stress factor. In *C. vulgaris* biofilms exposed to irradiances between 100 and 500 µmolm^−2^·s^−1^ for 15 days, the strongest reduction in F_v_/F_m_ was observed at 300 µmolm^−2^·s^−1^ (60%), followed by a 45% decrease at 500 µmol m^−2^ s^−1^ [63].

*Pseudokirchneriella subcapitata* is widely used in rapid toxicity assays to assess the effects of various environmental contaminants. In one study, measurements after 80 min of exposure revealed that herbicides, metribuzin (40 nM), atrazine (460 nM) and glyphosate (440 µM) caused reductions in maximum PSII activity of 8.3%, 13.9% and 9.0% respectively [65]. Herbicides typically impair green microalgae by targeting PSII, triggering oxidative stress, cellular damage and growth inhibition [75,76]. In contrast, 1 mM KCN did not produce a rapid or pronounced decline in F_v_/F_m_ [65]. The authors concluded that although KCN caused nearly complete suppression of F_v_/F_m_ in *P. subcapitata*, its effect developed more slowly than that of PSII-targeting herbicides. The slower response is attributed to the inhibition of mitochondrial cytochrome oxidase, which disrupts cellular energy metabolism and subsequently affects photosynthesis. This indirect mechanism, rather than direct damage to PSII, may explain the delayed and gradual decline in F_v_/F_m_. Furthermore, *P. subcapitata* was used for metal toxicity assessments, where cadmium (II), chromium (VI), copper (II) and zinc (II) were tested over a 72 h exposure period [66]. The most pronounced decline in F_v_/F_m_ (−84%) was observed with Cr(VI) at 41 µM, while at 2.7 µM, the effect was minimal. The next most significant reductions were induced by 1.3 µM Cu(II), followed by 2.5 µM Zn(II) and 1.9 µM Cd(II), with the respective F_v_/F_m_ decreases of 32%, 28% and 15% [66]. Additionally, the effects of endocrine-disrupting compounds were also evaluated using *P. subcapitata*. Both 4-octylphenol and β-estradiol exhibited minimal impact at concentrations of 5 ppm, whereas 4-nonylphenol at the same concentration led to a modest 2% reduction in F_v_/F_m_ [28]. Another study examined the influence of natural and synthetic humic substances on *P. subcapitata* and *Monoraphidium braunii* [64]. At low concentrations, these substances slightly enhanced the growth rate and maximum PSII activity, while concurrently reducing cell size and dry weight per cell. These changes were interpreted as adaptive responses to the presence of light-absorbing compounds in the medium. However, the authors also noted that the humic substances elicited more pronounced negative effects in other tested species, suggesting species-specific sensitivity to colored dissolved organic matter [64].

Research on the genus *Scenedesmus* has primarily focused on its responses to two categories of contaminants: pharmaceuticals and dissolved metals. In a study using *Scenedesmus obliquus* (with the current scientific name *Tetradesmus obliquus*), the effects of several common pharmaceuticals, including ketoprofen, aspirin, and both S-(+)- and racemic (rac)-ibuprofen, were assessed over exposure periods of 24, 48, 72, and 96 h. Ketoprofen exhibited the highest toxicity, followed by aspirin and racemic ibuprofen, while S-(+)-ibuprofen was found to be the least toxic compound [68]. The authors proposed that the studied pharmaceuticals impaired PSII efficiency in *S. obliquus* primarily by disrupting chloroplast structure, degrading photosynthetic pigments and damaging PSII reaction centers, which led to reduced light capture and electron transport. These structural effects were accompanied by downregulation of photosynthetic genes, reduced energy dissipation and decreased photosynthetic and respiratory rates, ultimately diminishing the alga’s photosynthetic performance [68]. In parallel, the *Scenedesmus* sp. YaA6, originally isolated from lead-contaminated waters, was investigated for its physiological responses to Pb exposure [77]. The study found that, while α (the initial slope of the P-I curve) remained unaffected at Pb concentrations as low as 0.87 nM, the maximum F_v_/F_m_ declined significantly, indicating differential sensitivity of photosynthetic parameters to Pb stress [77]. Elevated Pb concentrations in microalgal cultures further inhibited photosynthesis, largely through chlorophyll degradation [78]. Emerging studies have begun to explore the dual role of artificial sweeteners as both environmental contaminants and modulators of algal stress responses [67]. In *S. obliquus*, the presence of acesulfame enhanced the accumulation and tolerance of cells to Cu and Cd, indicating a potential protective effect [67]. Sucralose also showed a mild positive influence on Cu resistance but had a negligible impact on Cd toxicity. These findings suggest that some artificial sweeteners may modulate algal resilience to heavy metal stress, although their broader ecological implications require further investigation [67].

**Figure 2:**
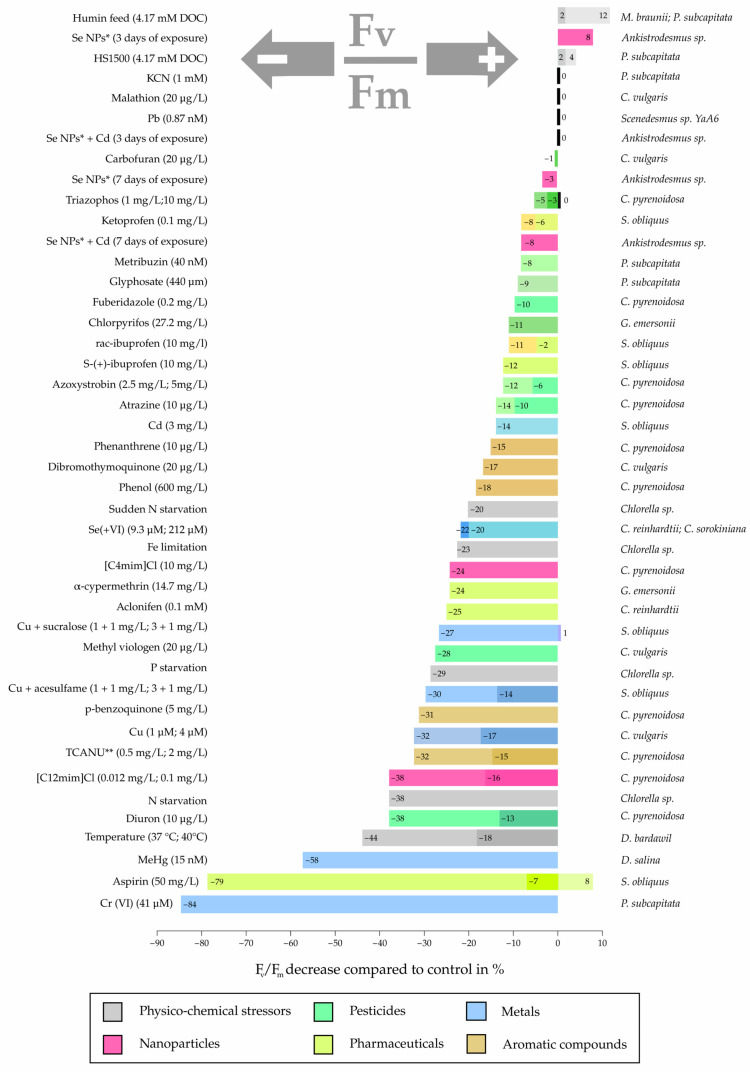
Comparison of the different stressors’ effects on the maximum PSII activity (F_v_/F_m_) in different microalgal species, as reported in the literature and Table 1. Notes: * Se nanoparticles; ** Trichloroacetonitrile uric acid.

**Figure 3:**
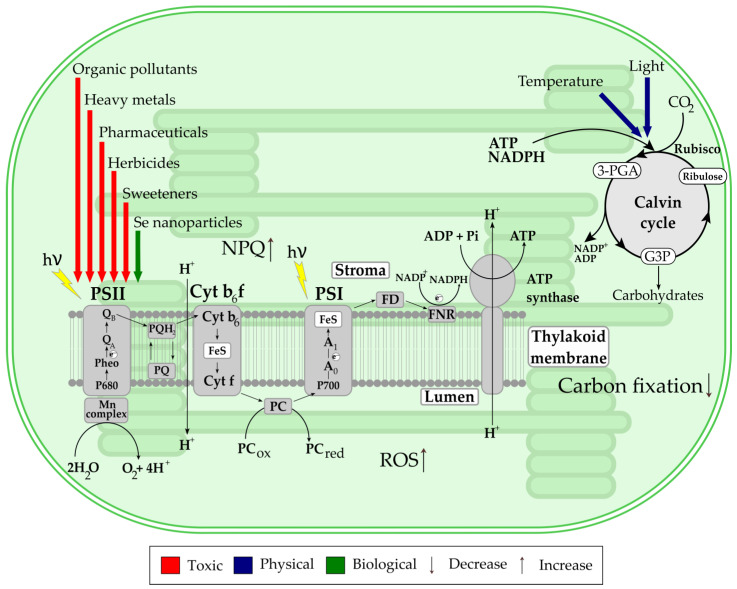
Mechanistic overview of the stressors’ impact on the photosynthetic apparatus of microalgae located in the chloroplast: photosynthetic thylakoid membrane and Calvin cycle (stroma). Abbreviations: P680: Reaction centers of PSII; P700: Reaction center of PSI; Cyt b_6_ and Cyt f: Cytochromes; FD: Ferredoxin; FeS: Rieske iron-sulfur proteins; FNR: Ferredoxin-NADP reductase; A_0_: primary electron acceptor of PSI; A_1_: phylloquinone molecule; PC: Plastocyanin; Pheo: Pheophytin; PQ: Plastoquinone; PQH_2_: Reduced plastoquinone; Q_A_: tightly bound plastoquinone; Q_B_: loosely bound plastoquinone; RuBP: ribulose-1,5-bisphosphate; 3-PGA: 3-phosphoglyceric acid: Rubisco: RuBP carboxylase/oxygenase enzyme; G3P: glyceraldehyde-3-phosphate; ROS: reactive oxygen species; NPQ: nonphotochemical quenching; Mn complex: water splitting complex (containing four Mn atoms), bound to PSII; NADP^+^: the oxidized form of nicotinamide adenine dinucleotide phosphate; NADPH: the reduced form of nicotinamide adenine dinucleotide phosphate; ATP: Adenosine triphosphate; ADP: Adenosine diphosphate.

Of the 44 different chemical and physiological stressors evaluated in microalgal cultures, only a few have solely positive (6.8%) effects on F_v_/F_m_, while 11.4% had a neutral or insignificant impact on photosynthetic efficiency compared to the control. The vast majority of stressors (84%) caused reductions in F_v_/F_m_, with the magnitude of inhibition depending on the stressor type and concentration (Figure 2). These data suggest that the chloroplast is the primary target of the majority of studied stressors, with the greatest effect on the thylakoid membrane, which further translates to the reaction centers, linear electron flow and overall PSII performance (Figure 3).

A mechanistic overview summarizing the hypothesized sites/modes of action and linking stressor classes to characteristic photosynthetic apparatus responses is presented in Figure 3.

Based on the available literature and the mechanistic overview presented in Figure 3, most chemical pollutants, such as herbicides or pharmaceuticals, have straight directional toxicity on PSII, but also affect PSI, through elevated oxidative stress and secondary PSI damage, which diminishes PSI ability to transfer electrons to NADP^+^ [79]. Although species of the genera *Chlorella*, *Scenedesmus* and *Pseudokirchneriella* dominate in ecotoxicological research, several less commonly studied microalgae have also provided valuable insights into species-specific responses to environmental stressors. For example, *Platymonas subcordiformis* was used to assess the ecotoxicological impacts of industrial chemical spills involving butyl acrylate [80]. Exposure to 10 mg/L of butyl acrylate resulted in a progressive reduction in F_v_/F_m_ of 4%, 7%, and 19% after 24, 48, and 96 h, respectively, indicating time-dependent inhibition of photosynthetic efficiency [80]. The authors proposed that butyl acrylate impaired photosynthesis by affecting the photochemical efficiency and electron transport within PSII, which led to a decrease in F_v_/F_m_. Additionally, exposure to butyl acrylate promoted NPQ, suggesting that part of the absorbed light energy was dissipated as heat to avoid photodamage, which is a common stress response in photosynthetic organisms. *Monoraphidium convolutum* has been studied for its response to chromate toxicity [22]. Concentrations exceeding 1 mg/L initially stimulated Φ_PSII_ during the first 2 h of exposure, followed by strong inhibition at 48 and 72 h. This biphasic response suggests that the antioxidant defenses may temporarily counteract oxidative damage at sublethal concentrations [22]. The authors attribute this to an early-stage overactivation of electron flow within PSII. This response likely reflects oxidative overstimulation of the thylakoid membrane before the onset of long-term damage. The authors further proposed that the primary site of Cr(VI) toxicity is the water-splitting complex on the oxidizing side of PSII, possibly via displacement of Mn^2+^ ions or oxidative damage to electron carriers, leading to higher ETR through the thylakoid membrane of the cells, resulting in increased reactive oxygen species (ROS) production [22]. The halotolerant microalga, *Dunaliella salina* (older name *Dunaliella bardawil*), was used to examine the relationship between heat stress and the expression of orange carotenoid-binding proteins [23]. In *D. salina*, orange protein is involved in carotenoid homeostasis and improves tolerance to environmental stress [23]. In contrast, photoprotection in cyanobacteria involves the orange carotenoid protein (OCP), which dissipates excess excitation energy by quenching phycobilisome fluorescence during high-light stress [81,82]. While OCP is a photoactive protein that senses light intensity and triggers photoprotection, its function is specific to cyanobacteria and it is not shared by orange protein in microalgae, which do not contribute to NPQ or thylakoid photoprotection [83,84]. The study demonstrated a temperature-dependent upregulation of specific stress-related genes when cultures were exposed to 40 °C, with full recovery observed within 24 h after short-term heat exposure. These findings highlight the thermal resilience and regulatory plasticity of this algal species [23]. Moreover, *D. salina* was strongly affected by the addition of 15 nM of methylmercury (MeHg), where F_v_/F_m_ values decreased by 58% during the 72 h of exposure [57]. Similarly, a significant decrease of Φ_PSII_ in *A. pyrenoidosa* added with 0.05–1.0 mg/L Hg^2+^ was reported [26]. High toxicity of Hg^2+^ to eukaryotic microalgae is attributed to its high affinity for thiol groups and reactivity with carboxyl, amide and amine groups [85,86]. Hg inhibits both light and dark phases of photosynthesis and can replace Mg in the chlorophyll molecule [87]. Authors reaffirmed the general opinion that the toxicity of heavy metals in photosynthesis is related to the binding abilities of metals to the photosynthetic apparatus and discovered the strong binding ability between Hg and the two photosystems [26]. The inhibition of PSII by Hg occurs mostly at its donor side. Hg primarily inhibits the donor side of PSII and reduces electron transport more strongly than in PSI [26]. It is noteworthy to emphasize that PAM fluorometry, while rapid and non-invasive, has limitations as it cannot fully distinguish species-specific responses or capture longer-term effects on algal growth and metabolism. In addition, results from laboratory monocultures may not directly reflect natural environments where multiple stressors act on diverse algal communities. The establishment of a reference database could therefore enhance data comparability and support species selection in future studies. Such a database would rely on defining baseline fluorescence values under strictly controlled conditions (temperature, light intensity, nutrient status) for representative microalgal species from each major genus, supported by well-characterized laboratory strains (e.g., *Chlorella vulgaris, Scenedesmus obliquus, Chlamydomonas reinhardtii*). The use of harmonized PAM measurement protocols, including dark adaptation time, actinic light intensity, and saturation pulse duration, following standardized reporting templates established in photosynthesis research would further improve data reproducibility. Despite these constraints, PAM fluorometry remains a valuable screening tool for detecting early stress responses and linking laboratory studies with environmental monitoring.

## 4. Conclusions

We can conclude that the effects of stressors on microalgal photosynthetic performance are almost always reflected in PSII activity and electron transport rate. Reported changes in F_v_/F_m_ range from: mildly positive (Se nanoparticles and humin feed); little or no effect (KCN (1 mM), Malathion (20 µg/L), Carbofuran (20 µg/L)); negative (15–20%) decrease (Atrazine (10 µg/L), Phenanthrene (10 µg/L), Phenol (600 mg/L), selenate (238.2 μM), Cd (3 mg/L)) to total impairment of the PSII and eventual culture death (Diuron (10 µg/L), MeHg (15 nM), Cr (VI) (41 µM), Aspirin (50 mg/L) and exposure to the high temperatures). These findings demonstrate that the impact of a stressor on photosynthetic efficiency depends not only on its chemical nature but also on its concentration and the species-specific sensitivity. We can also conclude that PAM fluorometry remains a reliable technique for rapidly assessing microalgal health and photosynthetic performance under diverse experimental conditions, as it is sensitive to a broad range of chemical and physical stressors. Nevertheless, PAM-derived parameters should be validated using complementary methods, such as the construction of the P-I curve based on oxygen evolution, to avoid potential errors in dark adaptation of the sample and subsequent PAM measurements.

## Data Availability

No new data were created or analyzed in this study. Data sharing is not applicable to this article.

## Abbreviations

The following abbreviations are used in this manuscript:

PAM	Pulse-amplitude modulated fluorimetry
PAR	Photosynthetically active radiation
F_v_/F_m_	PSII activity or maximum PSII activity
Φ_PSII_	Effective PSII activity of the Photosystem II
NPQ	Non-photochemical quenching
q_N_	Coefficient of non-photochemical quenching
q_P_	Coefficient of photochemical quenching
Q_A_	Primary electron acceptor of PSII
PPFD	Photosynthetic photon flux density
PSI	Photosystem I
PSII	Photosystem II
MeHg	Methylmercury
F_v_	Variable fluorescence
F_0_	Minimal (zero) fluorescence
F_m_	Maximal fluorescence
F	Fluorescence (chlorophyll)
α	Initial slope of photosynthesis−oxygen production (or RLC curve) equivalent to the F_v_/F_m_
OJIP	Fast fluorescence rise kinetics curve
F′_m_	Maximum fluorescence in the light-adapted state
F′	Steady-state fluorescence
F′_0_	Minimum fluorescence in the light-adapted state
σ_PSII_	Functional absorption cross section of PS II (nm^2^)
rETR	Relative electron transport rate
Ik	Saturating irradiance
RLCs	Rapid light response curves
FRRf	Fast repetition rate fluorometer
EC_50_	Concentration of the toxicant which induces 50% growth rate reduction
